# Linear analysis near a steady-state of biochemical networks: Control analysis, correlation metrics and circuit theory

**DOI:** 10.1186/1752-0509-2-44

**Published:** 2008-05-15

**Authors:** William J Heuett, Daniel A Beard, Hong Qian

**Affiliations:** 1 Laboratory of Biological Modeling, National Institute of Diabetes and Digestive and Kidney Diseases, National Institutes of Health, Bethesda, MD 20892, USA; 2 Biotechnology and Bioengineering Center and Department of Physiology, Medical College of Wisconsin, Milwaukee, WI 53226, USA; 3 Departments of Applied Mathematics and Bioengineering, University of Washington, Seattle, WA 98195, USA

## Abstract

**Background:**

Several approaches, including metabolic control analysis (MCA), flux balance analysis (FBA), correlation metric construction (CMC), and biochemical circuit theory (BCT), have been developed for the quantitative analysis of complex biochemical networks. Here, we present a comprehensive theory of linear analysis for nonequilibrium steady-state (NESS) biochemical reaction networks that unites these disparate approaches in a common mathematical framework and thermodynamic basis.

**Results:**

In this theory a number of relationships between key matrices are introduced: the matrix **A **obtained in the standard, linear-dynamic-stability analysis of the steady-state can be decomposed as **A **= **SR**^*T *^where **R **and **S **are directly related to the elasticity-coefficient matrix for the fluxes and chemical potentials in MCA, respectively; the control-coefficients for the fluxes and chemical potentials can be written in terms of **R**^*T*^**BS **and **S**^*T*^**BS **respectively where matrix **B **is the inverse of **A**; the matrix **S **is precisely the stoichiometric matrix in FBA; and the matrix *e*^**A***t *^plays a central role in CMC.

**Conclusion:**

One key finding that emerges from this analysis is that the well-known summation theorems in MCA take different forms depending on whether metabolic steady-state is maintained by flux injection or concentration clamping. We demonstrate that if rate-limiting steps exist in a biochemical pathway, they are the steps with smallest biochemical conductances and largest flux control-coefficients. We hypothesize that biochemical networks for cellular signaling have a different strategy for minimizing energy waste and being efficient than do biochemical networks for biosynthesis. We also discuss the intimate relationship between MCA and biochemical systems analysis (BSA).

## Background

Developing methodologies for quantitative analysis, both experimental and mathematical, of complex biochemical networks has become one of the central themes of post-genomic biochemistry and mathematical biology. Several disparate approaches, including metabolic control analysis (MCA) [1,2], flux balance analysis (FBA) [3,4], and correlation metric construction (CMC) [5,6], share many commonalities. The objective of this work is to provide a unifying mathematical framework and a thermodynamic basis for these approaches. The thermodynamic basis is the nonequilibrium steady-state (NESS) theory [7,8] originally developed to describe macromolecular, free-energy transduction [9-13], and the mathematical methods are based on linear analysis near a NESS.

The physical concept of a NESS (also known simply as a steady-state in the MCA community) applies to that of a driven system, such as a living organism, with a constant flow of matter (transforming nutrients to waste) through the system and a corresponding dissipation of free energy [9]. Even though a NESS looks remarkably similar to a thermodynamic equilibrium, in that chemical concentrations reach stationary values, the concentrations are maintained constant in a NESS by balancing influxes and effuxes, rather than by balancing the forward and backward fluxes of each elementary reaction as in thermodynamic equilibrium. To an observer concerned only with the chemical concentrations, a NESS would seem to be a true equilibrium; but, in fact, it represents a pseudo-equilibrium, where the work done to drive the system appears as heat, and deserves further consideration [9].

We distinguish between two types of linear analysis: (i) linear stability analysis [14], and (ii) control analysis (or sensitivity analysis) that addresses how the steady-state shifts in response to certain perturbations to the system. These perturbations may be deterministic–leading to MCA–or stochastic–leading to CMC. As expected, the matrix **A **obtained in linear stability analysis, the flux control-coefficient matrix **C **in MCA, the stoichiometric matrix **S **of FBA, and the correlation matrix **R **from CMC are intimately related. We develop the relationships in the context of complex biochemical networks and their NESS thermodynamics. We show that all the key matrices in MCA and CMC can be computed if one knows the fluxes, chemical potential differences, and the stoichiometric matrix.

The theoretical concept of a NESS provides valuable mathematical and thermodynamic properties. Although many of the approaches mentioned above have provided a mathematical treatment of NESS, many have never explicitly taken the related thermodynamics into account. The key insight is to consider concentrations as measures of potentials, providing a relationship between potential differences (voltages), fluxes (currents), and resistances. It becomes immediately clear that the resistances are related to reaction effectors, such as the expression levels of enzymes, that affect both the forward and backward fluxes of a given reaction, but do not change the chemical potential difference. It is this thermodynamic perspective of a NESS that can be used to develop a comprehensive theory that unites these approaches.

In the context of the newly developed biochemical circuit theory (BCT) [15,16], the flux *J *of each metabolic reaction in a NESS metabolic network is decomposed into *J *= *φ*^+ ^- *φ*^- ^and the chemical potential difference of the reaction in the NESS is Δ*μ *= *k*_*B*_*T *ln(*φ*_-_/*φ*_+_), where *k*_*B *_is Boltzmann's constant and *T *is absolute temperature. Therefore, knowing the chemical potential difference and the flux of a reaction gives

(1) $$\begin{array}{ccc} \varphi_{+}=\frac{J}{1-e^{\Delta \mu /k_{B}T}} & \text{and} & \varphi_{-}=\frac{Je^{\Delta \mu /k_{B}T}}{1-e^{\Delta \mu /k_{B}T}}. \end{array}$$

Furthermore, if the standard-state free energy of reaction Δ*μ*^*o *^is known from equilibrium thermodynamics, then $e^{(\Delta \mu -\Delta \mu^{o})/k_{B}T}$ determines the ratio of the metabolites' concentrations, products to reactants, in NESS.

In many theoretical approaches, metabolic networks and signaling pathways are often separated into disparate mechanisms with only peripheral interactions. This is far from reality, however, as these mechanisms are intimately intertwined and often indistinguishable. For example, in whole-body metabolism, glucose and fatty acids serve as the fuel for cellular metabolism, but also as signals for insulin secretion in pancreatic *β*-cells and ketogenesis in hepatocytes, where cellular organelles, such as mitochondria, play a key role in the handshake between metabolism and signaling. Ultimately, our goal is to understand how these cellular systems behave in response to stress and disease [17]. It is in applying the unified mathematical framework that includes the thermodynamic perspective to these processes that we believe the most significant contributions will be made.

In application, the concepts presented here allows one who has the stoichiometric matrix, **S**, and measures the correlation matrix, **R**, to obtain the control coefficients, and vice versa (Table 1). Furthermore, by incorporating the thermodynamic aspect, one is able to take full advantage of the thermodynamic properties, such as standard free energies of formation, which are typically more complete, more consistent, and more well analyzed than the kinetic information [17]. This is advantageous in a field where the availability of kinetic parameters is a limiting factor. However, available data remains incomplete, and missing thermodynamic and kinetic data must continually be obtained [17].

**Table 1:** Summary and comparison table listing the relationship between the elasticity and control coefficient matrices and the stoichiometric (S) and correlation (R) matrices, where A = SR^*T *^and B = A^-1^

**Symbol**	**Coefficient**	**Relationship**
***ε***	local elasticity	(dg **J**)^-1 ^**R**^*T*^
*ε*	steady-state elasticity	(dg **J**)^-1 ^**R**^*T*^**B **(dg **B**)^-1^
$\hat{C}$	concentration control	-**BS **(dg **J**)
**C**	flux control	**I **- (dg **J**)^-1 ^**R**^*T*^**BS **(dg **J**)
***ε***^Δ*μ*^	biochemical potential local elasticity	*k*_*B*_*T *(dg **Δ***μ*)^-1 ^**S**^*T*^
***ε***^Δ*μ*^	biochemical potential steady-state elasticity	*k*_*B*_*T *(dg Δ *μ*)^-1 ^**S**^*T*^**B **(dg **B**)^-1^
**C**^Δ*μ*^	biochemical potential control	-*k*_*B*_*T *(dg **Δ***μ*)^-1 ^**S**^*T*^**BS **(dg **J**)

### Basic kinetic equations for biochemical networks

Let us consider a network of *M *biochemical reactions involving *N *biochemical species *X*_*i *_(*i *= 1, 2, ..., *N*). The *j*th biochemical reaction (*j *= 1, 2, ..., *M*) is characterized by a set of stoichiometric coefficients $\nu =\{\nu_{i}^{j}\}$ and $\kappa =\{\kappa_{i}^{j}\}$ such that

(2) $$\nu_{1}^{j}X_{1}+\nu_{2}^{j}X_{2}+\cdots +\nu_{N}^{j}X_{N}\overset{k_{+}^{j}}{\underset{k_{-}^{j}}{⇌}}\kappa_{1}^{j}X_{1}+\kappa_{2}^{j}X_{2}+\cdots +\kappa_{N}^{j}X_{N}.$$

Some of the integer *ν*'s and *κ*'s can be zero. The *N *× *M *matrix **S **= ***κ ***- ***ν ***is known as the stoichiometric matrix. Eq. (2) assumes that the forward and backward reaction kinetics are characterized by the constants $k_{+}^{j}$ and $k_{-}^{j}$. In cases where the kinetic scheme involves intermediate steps (e.g., enzyme binding), each of the intermediate steps can be incorporated as reactions in the form of Eq. (2), requiring additional kinetic parameters, and the stoichiometric matrix can be constructed to include elementary reactions representing actual interaction events. Segel [18], for example, details how the Michaelis-Menten mechanism is expressed in terms of Eq. (2).

For the reaction system (2), kinetic equations of the time-dependent concentration changes for each biochemical species can be written according to the law of mass action as [19,20]

(3) $$\frac{dx_{i}(t)}{dt}=\sum_{j=1}^{M}\left(\kappa_{i}^{j}-\nu_{i}^{j}\right)\left(k_{+}^{j}x_{1}^{\nu_{1}^{i}}x_{2}^{\nu_{2}^{i}}\cdots x_{N}^{\nu_{N}^{j}}-k_{-}^{j}x_{1}^{\kappa_{1}^{j}}x_{2}^{\kappa_{2}^{j}}\cdots x_{N}^{\kappa_{N}^{j}}\right)+J_{i}^{e},$$

where we use *x*_*ℓ*_to denote the concentration of respective *X*_*ℓ*_, and $J_{i}^{e}$ (*i *= 1, 2, ..., *N*) are external injection fluxes to species *i *(also known as boundary fluxes). We use the convention that subscripts index species and superscripts index reactions. To further simplify the notation, we introduce forward and backward fluxes $J^{j}=\varphi_{+}^{j}-\varphi_{-}^{j}$[20]:

$$\begin{array}{c} \varphi_{+}^{j}=k_{+}^{j}{\left(x_{1}\right)}^{\nu_{1}^{j}}{\left(x_{2}\right)}^{\nu_{2}^{j}}\cdots {\left(x_{N}\right)}^{\nu_{N}^{j}},\\ \varphi_{-}^{j}=k_{-}^{j}{\left(x_{1}\right)}^{\kappa_{-}^{j}}{\left(x_{2}\right)}^{\kappa_{2}^{j}}\cdots {\left(x_{N}\right)}^{\kappa_{N}^{j}}. \end{array}$$

Then Eq. (3) becomes *d*x/*dt *= **SJ **+ **J**^*e*^, in which **x **and **J**^*e *^are *N*-dimensional column vectors, and **J **is an *M*-dimensional column vector.

In a ***closed ***reaction system, **J**^*e *^= **0**. In this case, it can be shown that thermodynamic equilibrium is the only positive stationary solution to Eq. (3): the internal fluxes **J **= ***φ***_+ _- ***φ***_- _= **0 **for each and every reaction. For a closed biochemical reaction system, since all the fluxes are necessarily zero in its unique equilibrium (for each given set of parameters; unique in the dynamic sense), all the control coefficients are necessarily zero. Therefore, in a NESS, at least one injection flux and one effux are nonzero ($J_{i}^{e}$ ≠ 0), or certain concentrations *x*_*i *_are held at constant levels. The former case is referred to as "external flux injection," while the latter is referred to as "external concentration clamping." A distinction between these two "forcing" mechanisms greatly clarifies many controversial issues concerning NESS.

### Steady-state concentrations

In the equilibrium state of a closed reaction system, ***φ***_+ _= ***φ***_- _and the ratio of chemical concentrations is independent of the amount of material and the initial condition:

$$\frac{{\left(x_{1}\right)}^{\kappa_{-}^{j}}{\left(x_{2}\right)}^{\kappa_{2}^{j}}\cdots {\left(x_{N}\right)}^{\kappa_{N}^{j}}}{{\left(x_{1}\right)}^{\nu_{1}^{j}}{\left(x_{2}\right)}^{\nu_{2}^{j}}\cdots {\left(x_{N}\right)}^{\nu_{N}^{j}}}=\frac{k_{+}^{j}}{k_{-}^{j}},$$

giving rise to the concept of chemical equilibrium constants. Such a system of biochemical reactions is non-dissipative and is associated with a number of conserved quantities. An essential mathematical characteristic of the closed system is that its equilibrium point is degenerate (neutrally stable on a certain manifold), i.e., there is no unique stationary solution for different initial conditions.

For an open system that approaches a NESS, the stationary solution is not usually degenerate. If we assume {$x_{i}^{*}$} is an asymptotically stable NESS, small differences in the initial condition should lead to the ***same ***NESS. We use the notations S to denote the set of concentrations of internal species and I to denote the concentrations of external species which are clamped or independently varied.

In the linear regime near the NESS [17],

(4) $$\frac{d}{dt}(\delta x_{i})=\sum_{j=1}^{N}A_{ij}\frac{\left(\delta x_{j}\right)}{x_{j}^{*}},$$

in which *δx*_*j *_= *x*_*j *_- $x_{j}^{*}$ and

$$A_{ij}=\sum_{\ell =1}^{M}\left(\kappa_{i}^{\ell }-\nu_{i}^{\ell })(\nu_{j}^{\ell }\varphi_{+}^{\ell }-\kappa_{j}^{\ell }\varphi_{-}^{\ell }\right).$$

**A **= {*A*_*ij*_} is called the linear stability matrix. We introduce the *N *× *M *matrix **R **= $\left\{R_{ij}=\nu_{i}^{j}\varphi_{+}^{j}-\kappa_{i}^{j}\varphi_{-}^{j}\right\}$, such that **A **= **SR**^*T*^. We also denote **A**^-1 ^= **B**.

If the nonlinear dynamics scheme of Eq. (3) conserves certain quantities, such as total element, motif, or enzyme concentrations, **S **does not have full rank and **A **is singular [17,19,20]. In such cases, it is possible to transform **A **into a nonsingular matrix by replacing its linearly dependent rows with vectors in its left null space (see Ref. 18 of [20]). By doing so, one removes the redundancies from the original dynamics scheme. A more detailed mathematical analysis will be published elsewhere. Both here and below, **A **is understood to be the transformed nonsingular matrix.

## Results and Discussion

### Metabolic control analysis

Eq. (3) is a general scheme for biochemical reaction networks. For metabolic reactions involving enzymes, the enzyme and the enzyme-substrate complexes are treated as additional "species." In this section, however, we study MCA, and assume that every reaction involves an enzyme which is not explicitly expressed as a species. Rather, we assume enzyme activity is absorbed into the rate constants for the forward and backward reactions. This is clearly not an accurate approximation for many enzymatic reaction mechanisms. Nevertheless, it provides a first-order approximation for treating a metabolic network. More complex rate laws for enzymatic reactions have been discussed for biochemical systems analysis [21,22].

MCA focuses on how the NESS {$x_{i}^{*}$} shifts in response to a perturbation to the amount of enzyme for a reaction or a perturbation to its substrate (∈ I) concentration. Without losing generality, we assume these perturbations are made to the enzyme for the M*th *reaction or the N*th *(external) species.

#### Elasticity coefficients

First, we consider the case where the concentration of external species *N *is changed: $x_{N}^{*}\to x_{N}^{*}+\delta x_{N}$. Before reaching a new NESS, the local response is only in the flux of the reactions involving *X*_*N*_. The immediate, local change is characterized by

(5) $$\varepsilon_{N}^{m}≜\frac{x_{N}^{*}}{J^{m}}\left(\frac{\delta J^{m}}{\delta x_{N}}\right)=\frac{\nu_{N}^{m}\varphi_{+}^{m}-\kappa_{N}^{m}\varphi_{-}^{m}}{\varphi_{+}^{m}-\varphi_{-}^{m}}=\frac{R_{Nm}}{J^{m}}$$

which is called the scaled, local elasticity-coefficient [1]. These coefficients are elements of the scaled, local, elasticity-coefficient matrix, ***ε ***= (dg **J**)^-1 ^**R**^*T*^. Here, we use the same notation used by Heinrich and Schuster [23], where (dg **v**) denotes the diagonal matrix containing the components of the vector **v **along its diagonal. The unscaled, local, elasticity-coefficient matrix is given by *ε' *= (dg **J**) *ε *(dg x)^-1 ^= **R**^*T *^(dg x)^-1^. The prime symbol is used to distinguish unscaled coefficients from scaled coefficients, both here and in what is to follow.

The coefficient *ε *should be set apart from the coefficient *ε*, which characterizes the steady-state response [24]. When a new NESS is established, the concentrations {*x*_*i*_} (*i *= 1, 2, ..., *N *- 1) satisfy

(6) $$\sum_{j=1}^{N-1}A_{ij}\frac{\delta x_{j}}{x_{j}^{*}}=-A_{iN}\frac{\delta x_{N}}{x_{N}^{*}}.$$

Solving Eq. (6), we have $\delta x_{i}/x_{i}^{*}=B_{iN}\delta x_{N}/B_{NN}x_{N}^{*}$ where *B*_*iN *_is the N*th *column vector of the matrix **A**^-1^. The new NESS established near {$x_{i}^{*}$} is {$x_{i}^{*}+\delta x_{i}$}, and the new fluxes, *J*^*m *^+ *δJ*^*m*^, are given by

(7) $$\begin{array}{ccc} \delta J^{m}=\delta \varphi_{+}^{m}-\delta \varphi_{-}^{m} & = & \sum_{\ell =1}^{N}(\nu_{\ell }^{m}\varphi_{+}^{m}-\kappa_{\ell }^{m}\varphi_{-}^{m})\frac{\delta x_{\ell }}{x_{\ell }^{*}}\\ & = & \sum_{\ell =1}^{N}(\nu_{\ell }^{m}\varphi_{+}^{m}-\kappa_{\ell }^{m}\varphi_{-}^{m})\frac{B_{\ell N}\delta x_{N}}{B_{NN}x_{N}^{*}}. \end{array}$$

Hence, the scaled, steady-state elasticity-coefficient is [17]

(8) $$\varepsilon_{N}^{m}≜\frac{x_{N}^{*}}{J^{m}}\left(\frac{\delta J^{m}}{\delta x_{N}}\right)=\frac{\sum_{\ell =1}^{N}(\nu_{\ell }^{m}\varphi_{+}^{m}-\kappa_{\ell }^{m}\varphi_{-}^{m})B_{\ell N}}{(\varphi_{+}^{m}-\varphi_{-}^{m})B_{NN}},$$

which may also be known as a response coefficient [23,25]. These coefficients are elements of the scaled, steady-state, elasticity-coefficient matrix, *ε *= (dg **J**)^-1 ^**R**^*T*^**B **(dg **B**)^-1^. Here, (dg **B**) denotes the diagonal matrix containing only the diagonal components of the matrix **B **along its diagonal. The unscaled, steady-state, elasticity-coefficient matrix is given by *ε' *= (dg **J**) *ε *(dg **x**)^-1 ^= **R**^*T*^**B **(dg **B**)^-1 ^(dg **x**)^-1^.

As we shall show, some connectivity theorems for the two quantities *ε *and *ε *are different. In principle, the *ε *can be experimentally measured as a system response, but obtaining *ε *can be more difficult since it is a transient response that must be measured individually.

#### Control coefficients

Next, we consider the case where the enzyme for the M*th *reaction *E*^*M *^is changed: *E*^*M *^→ *E*^*M *^+ *δE*^*M*^. Since we have assumed that the rate constants for reaction *M*, $k_{\pm }^{M}$, are linearly proportional to the concentration of the enzyme catalyzing reaction *M*, *E*^*M*^, we have

$$\frac{E^{M}}{k_{+}^{M}}\left(\frac{\delta k_{+}^{M}}{\delta E^{M}}\right)=\frac{E^{M}}{k_{-}^{M}}\left(\frac{\delta k_{-}^{M}}{\delta E^{M}}\right)=1.$$

When *E*^*M *^→ *E*^*M *^+ *δE*^*M*^, the new NESS satisfies [17]

(9) $$\sum_{j1}^{N}A_{ij}\frac{\delta x_{j}}{x_{j}^{*}}=(\nu_{i}^{M}-\kappa_{i}^{M})(\varphi_{+}^{M}-\varphi_{-}^{M})\frac{\delta E^{M}}{E^{M}}.$$

Solving for *δx*_*j*_/$x_{j}^{*}$ (*j *= 1, 2, ..., *N*), we have

(10) $$\frac{\delta x_{j}}{x_{j}^{*}}\sum_{j=1}^{N}B_{ji}=(\nu_{i}^{M}-\kappa_{i}^{M})(\varphi_{+}^{M}-\varphi_{-}^{M})\frac{\delta E^{M}}{E^{M}}.$$

From Eq. (10), we get the scaled, concentration control-coefficient [26],

(11) $${\hat{C}}_{M}^{m}≜\frac{E^{M}}{x_{j}^{*}}\left(\frac{\delta x_{j}}{\delta E^{M}}\right)=-\sum_{j=1}^{N}B_{ji}=(\kappa_{i}^{M}-\nu_{i}^{M})(\varphi_{+}^{M}-\varphi_{-}^{M}),$$

which is an element of the scaled, concentration, control-coefficient matrix given by $\hat{C}$ = -**BS**(dg **J**). The unscaled, concentration, control-coefficient matrix is ${\hat{C}}^{\prime }$ = (dg **x**) $\hat{C}$ (dg **J**)^-1 ^= -(dg **x**)**BS**.

Combining Eqs. (7) and (10), we get the scaled, flux control-coefficient [1],

(12) CjM≜EMJm(δJmδEM)=δmM−∑ℓ=1N∑i=1N(νℓmφ+m−κℓmφ−m)Bℓi(κiM−νiM)(φ+M−φ−M)(φ+m−φ−m),

where *δ*_*mM *_is the Kronecker delta function. These coefficients are elements of the scaled, flux, control-coefficient matrix given by **C **= **I **- (dg **J**)^-1 ^**R**^*T*^**BS **(dg **J**). The unscaled, flux, control-coefficient matrix is **C' **= (dg **J**) **C **(dg **J**)^-1 ^= **I **- **R**^*T*^**BS**.

#### Summation and connectivity theorems

Among *ε*, *ε*, *C*, and $\hat{C}$, we have the following relationships. First, note the relationships

(13) $$\hat{C}=-{\left(S(\text{dg }J)\varepsilon \right)}^{-1}\;S(\text{dg }J),$$

(14) $$C=I+\varepsilon \hat{C},$$

between the scaled coefficients, and similarly,

(15) $${\hat{C}}^{\prime }=-{\left(S\varepsilon^{\prime }\right)}^{-1}S,$$

(16) $$C^{\prime }=I+\varepsilon {\hat{\mathrm{\prime C}}}^{\prime },$$

between the unscaled coefficients.

Second, from Eqs. (13)–(16), it is straightforward to show that if there is no injection flux, i.e., in NESS **SJ **= -**J**^*e *^= 0, we have

(17) $$\hat{C}1_{M\times 1}=0_{N\times 1},$$

(18) **C1**_*M *× 1 _= 1_*M *× 1_,

where **1**_*i *× *j *_and **0**_*i *× *j *_denote the *i *× *j *matrices of ones and zeros, respectively. Eqs. (17) and (18) are known as the summation theorems for the scaled control-coefficients. For the unscaled control-coefficients, we have the generalized summation theorems, **C'K **= **K **and ${\hat{C}}^{\prime }K=0_{N\times (M-N)}$, where **K **is the *M *× (*M *- *N*) loop (or nullspace) matrix, i.e., its columns form a basis of the nullspace of **S **so that **SK **= **0**_*N *× (*M *- *N*) _[15,16].

Euler's theorem on homogeneous functions can be used to understand the significance of the injection fluxes [17,26]. If there are no injection fluxes and a NESS is sustained by clamped concentrations, the steady-state fluxes are homogeneous functions of the enzyme concentrations with order 1. That is to say that, assuming every reaction in the system is catalyzed by an enzyme, if every enzyme concentration is simultaneously doubled, the flux in each reaction doubles. This leads to the summation theorem for the flux control-coefficients. On the other hand, if there are no clamped concentrations and a NESS is sustained by injection fluxes, the steady-state fluxes are homogeneous functions of enzyme concentrations with order 0. A similar argument exists for steady-state concentrations as a function of enzyme concentrations, leading to the summation theorem for the concentration control-coefficients.

Third, **BSR**^*T *^= **I **⇒ (**R**^*T*^**BS**)(**R**^*T*^**B**) = **R**^*T*^**B**, which yields the connectivity theorems for the scaled coefficients:

(19) $$\begin{array}{cc} \hat{C}\varepsilon =-I, & \hat{C}\varepsilon =-B{\left(\text{dg }B\right)}^{-1}, \end{array}$$

(20) **C*ε ***= **0**_*M *× *N*_,   **C*ε ***= **0**_*M *× *N*_,

and the generalized connectivity theorems for the unscaled coefficients [23]:

(21) $$\begin{array}{cc} {\hat{C}}^{\prime }\varepsilon^{\prime }=-I, & {\hat{C}}^{\prime }\varepsilon^{\prime }=-(\text{dg }x)\;B{\left(\text{dg }B\right)}^{-1}{\left(\text{dg }x\right)}^{-1}, \end{array}$$

(22) **C*'ε' ***= **0**_*M *× *N*_,   **C*'ε' ***= **0**_*M *× *N*_.

Finally, by combining the generalized summation and connectivity theorems, we get

(23) $$\left(\begin{array}{c} C^{\prime }\\ {\hat{C}}^{\prime } \end{array}\right)\left(\begin{array}{cc} K & \varepsilon^{\prime } \end{array}\right)=\left(\begin{array}{ll} K & 0_{M\times N}\\ 0_{N\times (M-N)} & -I_{N\times N} \end{array}\right),$$

which is the central equation in MCA [23]. The matrix (**K *ε'***) has been proved to be invertible [23,27]. Therefore, if the local elasticity-coefficients are known, the control-coefficients can be calculated.

Using the steady-state elasticity-coefficients, we have

(24) $$\left(\begin{array}{c} C^{\prime }\\ {\hat{C}}^{\prime } \end{array}\right)\left(\begin{array}{cc} K & \varepsilon^{\prime } \end{array}\right)=\left(\begin{array}{ll} K & 0_{M\times N}\\ 0_{N\times (M-N)} & -(\text{dg }x)\;B{\left(\text{dg }B\right)}^{-1}{\left(\text{dg }x\right)}^{-1} \end{array}\right).$$

The matrix (**K ***ε'*) is also invertible (see Methods). Therefore, if the steady-state elasticity-coefficients are known, the control-coefficients can be calculated. This is an important result because ε can be experimentally measured as a system response, but obtaining ε can be more difficult since it is a transient response and must be measured individually.

### Biochemical potential and its control analysis

The most notable difference between BCT and other theories of biochemical networks is its sound thermodynamics basis [15,16]. BCT articulates the fluxes *J *and chemical potential differences Δ*μ *as two, complementary, key characteristics of a reaction in a NESS. Kirchhoff's flux and potential laws are manifestations of the fundamental laws of physical chemistry, namely conservation of mass and conservation of energy [15,16].

Both *J *and Δ*μ *can be written in terms of the forward and backward fluxes *φ*_+ _and *φ*_- _as given in Eq. (1), and vice versa. In general, the flux *J *and the chemical potential difference Δ*μ *are nonlinearly related [28]:

(25) $$J=\varphi_{+}\left(1-e^{\Delta \mu /k_{B}T}\right)=\varphi_{-}\left(e^{-\Delta \mu /k_{B}T}-1\right).$$

In the linear regime with small flux and chemical potential difference, *J *and Δ*μ *are linearly related with the biochemical conductance being *φ*_+_/*k*_*B*_*T *(= *φ*_-_/*k*_*B*_*T*) [11,16]. In the nonlinear regime, if a perturbation on a reaction is from its upstream, we can assume the *φ*_- _is relatively unperturbed. Then we have *δJ*/*δ*Δ*μ *= -*φ*_+_/*k*_*B*_*T*. Similarly, if the reaction is perturbed from its downstream, then *δJ*/*δ*Δ*μ *= -*φ*_-_/*k*_*B*_*T*.

Combining BCT and MCA, we can define the *scaled, local elasticity-coefficients for chemical potentials *[25]

(26) $${\left(\varepsilon^{\Delta \mu }\right)}_{n}^{m}≜\frac{x_{n}^{*}}{\Delta \mu^{m}}\left(\frac{\delta \Delta \mu^{m}}{\delta x_{n}}\right)=k_{B}T\frac{\left(\kappa_{n}^{m}-\nu_{n}^{m}\right)}{\Delta \mu^{m}}$$

as elements of the matrix ***ε***^Δ*μ *^= *k*_*B*_*T *(dg **Δ***μ*)^-1 ^**S**^*T*^, *the scaled, steady-state elasticity-coefficients for chemical potentials*

(27) $${\left(\varepsilon^{\Delta \mu }\right)}_{n}^{m}≜\frac{x_{n}^{*}}{\Delta \mu^{m}}\left(\frac{\delta \Delta \mu^{m}}{\delta x_{n}}\right)=k_{B}T\frac{\sum_{\ell =1}^{N}\left(\kappa_{\ell }^{m}-\nu_{\ell }^{m}\right)B_{\ell n}}{\Delta \mu^{m}B_{nn}}$$

as elements of the matrix *ε*^Δ*μ *^= *k*_*B*_*T *(dg **Δ***μ*)^-1 ^**S**^*T*^**B **(dg **B**)^-1^, and the *scaled, potential control-coefficients *[25]

(28) (CΔμ)nm≜EnΔμm(δΔμmδEn)=−kBT∑ℓ=1N∑i=1N(κℓm−νℓm)Bℓi(κin−νin)(φ+n−φ−n)Δμm

as elements of the matrix **C**^Δ*μ *^= -*k*_*B*_*T *(dg **Δ***μ*)^-1 ^**S**^*T*^**BS **(dg **J**), where reaction potentials and substrate concentrations in the reference state are used for normalization. The corresponding unscaled coefficients are given in matrix form as

(***ε***^Δ*μ*^)' = (dg **Δ***μ*) ***ε***^Δ*μ *^(dg **x**)^-1 ^= *k*_*B*_*T***S**^*T *^(dg **x**)^-1^,

(***ε***^Δ*μ*^)' = (dg **Δ***μ*) ***ε***^Δ*μ *^(dg **x**)^-1 ^= *k*_*B*_*T***S**^*T*^**B **(dg **B**)^-1 ^(dg **x**)^-1^,

(**C**^Δ*μ*^)' = (dg **Δ***μ*) **C**^Δ*μ *^(dg **J**)^-1 ^= -*k*_*B*_*T***S**^*T*^**BS**.

Note the appearance of the matrices **S**^*T*^, **S**^*T*^**B**, and **S**^*T*^**BS **in the local and steady-state elasticity-coefficients and the control-coefficient for the potentials, as compared to the matricies **R**^*T*^, **R**^*T*^**B**, and **R**^*T*^**BS **in the coefficients for the fluxes. Furthermore, note the relationships

(29) $$\hat{C}\{\Delta \mu =\varepsilon^{\Delta \mu }\hat{C},$$

(30) $$(\hat{C}\{\Delta \mu {)}^{\prime }=(\varepsilon^{\Delta \mu }{)}^{\prime }{\hat{C}}^{\prime }.$$

Eqs. (19) and (29) in combination yield the summation [25] and connectivity theorems for the scaled coefficients, under consideration of the steady-state condition,

(31) **C**^Δ*μ*^**1**_*M *× 1 _= **0**_*M *× 1_,

(32) **C**^Δ*μ*^***ε ***= -***ε***^Δ*μ*^,   **C**^Δ*μ*^*ε *= -**ε**^Δ*μ*^.

Eq. (31) can also be derived using the homogeneous function theory (discussed above with flux and concentration control-coefficients). For the unscaled coefficients, we get the generalized summation and connectivity theorems by combining Eqs. (21) and (30) so that

(33) (**C**^Δ*μ*^)' **K **= **0**_*M *× (*M *- *N*)_,

(34) (**C**^Δ*μ*^)' ***ε' ***= -(***ε***^Δ*μ*^)',   (**C**^Δ*μ*^)' ***ε' ***= -(***ε***^Δ*μ*^)'.

Eq. (33) is a manifestation of Kirchhoff's loop law [15,16].

By combining the generalized summation and connectivity theorems, we get

(35) $$\left(\begin{array}{c} (C^{\Delta \mu }{)}^{\prime }\\ {\hat{C}}^{\prime } \end{array}\right)\left(\begin{array}{cc} K & \varepsilon^{\prime } \end{array}\right)=\left(\begin{array}{ll} 0_{M\times (M-N)} & -(\varepsilon^{\Delta \mu }{)}^{\prime }\\ 0_{N\times (M-N)} & -I \end{array}\right).$$

The matrix (**K ***ε'*) has been proved to be invertible [23,27]. Therefore, if the local elasticity-coefficients are known, the control-coefficients can be calculated. Using the steady-state elasticity-coefficients, we have

(36) $$\left(\begin{array}{c} (C^{\Delta \mu }{)}^{\prime }\\ {\hat{C}}^{\prime } \end{array}\right)\left(\begin{array}{cc} K & \varepsilon^{\prime } \end{array}\right)=\left(\begin{array}{ll} 0_{M\times (M-N)} & -(\varepsilon^{\Delta \mu }{)}^{\prime }\\ 0_{N\times (M-N)} & -(\text{dg }x)\;B{\left(\text{dg }B\right)}^{-1}{\left(\text{dg }x\right)}^{-1} \end{array}\right).$$

The matrix (**K ***ε'*) has an inverse (see Methods). Therefore, if the steady-state elasticity-coefficients are known, the control-coefficients can be calculated.

### Correlation metric construction

Correlation metric construction (CMC) focuses on how the NESS {$x_{i}^{*}$} fluctuates in response to a random stochastic perturbation to the concentration level of an external species *x*_*N *_∈ I. We assume the perturbation is small. Hence, the linear analysis is appropriate.

If the random perturbation has a sufficiently long correlation time compared to the relaxations of all the reactions in the system, then we are dealing with a shift in the NESS where, according to Eq. (6),

$$\delta x_{i}/x_{i}^{*}=B_{iN}\delta x_{N}/B_{NN}x_{N}^{*}.$$

From the time series of {*δx*_*i*_} with an uncorrelated perturbation on *δx*_*N*_, we have

(37) $$\frac{\langle \delta x_{i}\delta x_{j}\rangle }{\sqrt{\langle {\left(\delta x_{i}\right)}^{2}\rangle \langle {\left(\delta x_{j}\right)}^{2}\rangle }}=1$$

for *i, j *= 1, 2, ..., *N*.

CMC extracts information from reaction systems by using perturbations with insufficiently long correlation times. *δx*_*N *_is a Markov process characterized by

(38) $$\frac{d}{dt}(\delta x_{N})=-k_{d}\delta x_{N}+a\xi (t),$$

where *k*_*d *_controls the relaxation time of the perturbation, *a *is its random amplitude with Gaussian distribution, and ξ(*t*) is a uncorrelated noise. *δx*_*N *_in Eq. (38) is called an Ornstein-Uhlenbeck (OU) process with auto-covariance function

(39) $$g(\tau )≜\langle \delta x_{N}(t)\delta x_{N}(t+\tau )\rangle =\frac{a^{2}}{2k_{d}}e^{-k_{d}|\tau |}.$$

With this type of random perturbation, the temporal correlations between the concentration fluctuations give information on the connectivity of a network following the kinetic equation (4)

(40) $$\frac{d}{dt}(\delta x_{i})=\sum_{j=1}^{N-1}\left(\frac{A_{ij}}{x_{j}^{*}}\right)\delta x_{j}+\frac{A_{iN}}{x_{N}^{*}}\delta x_{N}(t)$$

for *i *= 1, 2, ...,(*N *- 1). More precisely (A. Arkin, personal communication), instead of Eq. (40) CMC follows an autoregressive (AR) process, the discrete version of an OU process, with discrete time *T*, 2*T*, ...,

$$\delta x_{i}(n+1)=\sum_{i=1}^{N}{\left(e^{\tilde{\tilde{A}}T}\right)}_{ij}\delta x_{j}(n)-B_{iN}a\xi_{n},$$

in which ${\tilde{\tilde{A}}}_{ij}=A_{ij}/x_{j}^{*}-\delta_{iN}k_{d}$. The discrete AR and continuous OU models give essentially the same result.

Let's rewrite the **A **matrix into a (*N *- 1) × (*N *- 1) matrix $\tilde{A}=\{{\tilde{A}}_{ij}=A_{ij}/x_{j}^{*},i,j=1,2,...,N-1\}$. Then the stationary solution to Eqs. (38) and (40) is

(41) $$\delta x_{i}(t)=\sum_{\ell =1}^{N-1}\int_{0}^{t}{\left(e^{\tilde{A}(t-\eta )}\right)}_{i\ell }\frac{A_{\ell N}}{x_{N}^{*}}\delta x_{N}(\eta )d\eta ,$$

which leads to

(42) $$〈\delta x_{i}(t)\delta x_{j}(t+\tau )〉=\sum_{\ell ,m=1}^{N-1}\int_{0}^{\infty }\int_{0}^{\infty }{\left(e^{\tilde{A}\eta }\right)}_{i\ell }\frac{A_{\ell N}A_{mN}}{x_{N}^{*}x_{N}^{*}}{\left(e^{\tilde{A}\zeta }\right)}_{jm}g(\tau +\eta -\zeta )d\eta d\zeta .$$

Two limits of Eq. (42) are particularly interesting. If the correlation time of *g*(*τ*) in Eq. (39) is ≫ all the relaxation times of $\tilde{A}$, then we have Eq. (37). On the other hand, if the correlation time for the random perturbation is ≪ all the relaxation times of $\tilde{A}$, we have

(43) $$〈\delta x_{i}(t)\delta x_{j}(t+\tau )〉=\sum_{\ell =1}^{N-1}\langle \delta x_{i}\delta x_{\ell }\rangle {\left(e^{\tilde{A}r}\right)}_{j\ell }.$$

The matrix $R(t)=e^{\tilde{A}t}$ is the fundamental solution to the linear kinetic equation (4). *R*_*ij*_(*t*) is the response curve, as a function of *t*, for the *i*th species to an impulse of the *j*th species at time 0. The relation between the correlation matrix and the fundamental solution is expected for a linear system. For directly connected species *i *and *j*, the ${R^{\prime }}_{ij}(0)\neq 0$, whereas if *i *and *j *are not directly connected, ${R^{\prime }}_{ij}(0)=0$. Analytic solutions for completely linear systems have been derived and analyzed [29].

## Conclusion

### Rate limiting step: largest flux control-coefficient and smallest biochemical conductance

Both flux control-coefficients and biochemical conductances can be used as indicators for rate-limiting steps in a biochemical network [30]. Traditionally the rate-limiting step is understood in terms of the highest "activation barrier" in the pathway. Here we use a simple example to demonstrate that these concepts are intimately related. Mathematically, the highest activation barrier is also related to the mean first-passage time in stochastic dynamics [31,32].

Let's consider the linear pathway

(44) $$X_{0}\overset{k_{1}}{\underset{k-1}{⇌}}X_{1}\overset{k_{2}}{\underset{k-2}{⇌}}X_{2}\overset{k_{3}}{\underset{k-3}{⇌}}\cdots ⇌X_{n-2}\overset{k_{n-1}}{\underset{k_{-(n-1)}}{⇌}}X_{n-1}\overset{k_{n-1}}{\underset{k_{-n}}{⇌}}X_{n}$$

under NESS with clamped concentrations *x*_0 _and *x*_*n *_for *X*_0 _and *X*_*n*_, respectively. Analogous to a continuous energy landscape, we have the discrete energy function

$$E_{j}=-k_{B}T\ln\left(\frac{k_{1}k_{2}\cdots k_{j}}{k_{-1}k_{-2}\cdots k_{-j}}\right),$$

for *j *= 1, 2,..., *n*. If there is a rate-limiting step, say for example that reaction *j *is nearly irreversible, then *E*_*j *_is maximal and that is where the highest energy barrier is located. We now show that it is also where the largest flux control-coefficient and the smallest conductance are.

The NESS flux of reaction system (44) can be solved for analytically to yield [31,32]

(45) $$J=\frac{\frac{k_{1}k_{2}\cdots k_{n}}{k_{-1}k_{-2}\cdots k_{-n}}x_{0}-x_{n}}{\frac{1}{k_{-n}}+\frac{k_{n}}{k_{-n}k_{-(n-1)}}+\frac{k_{n}k_{n-1}}{k_{-n}k_{-(n-1)}k_{-(n-2)}}+\cdots +\frac{k_{n}k_{n-1}\cdots k_{2}}{k_{-n}k_{-(n-1)}\cdots k_{-1}}}.$$

Therefore, the flux control-coefficient is

(46) CℓJ=EℓJ(δJδEℓ)=knkn−1⋯kℓ+1k−nk−n(n−1)⋯k−(ℓ+1)k−ℓ1k−n+knk−nk−(n−1)+knkn−1k−nk−(n−1)k−(n−2)+⋯+knkn−1⋯k2k−nk−(n−1)⋯k−1,

which reaches its maximum at maximal *E*_*j*_. The biochemical conductance, on the other hand, is

$$\left|\frac{J}{\Delta \mu_{\ell }}\right|=\frac{J}{k_{B}T}{\left(\ln\frac{\varphi_{+}^{\ell }}{\varphi_{+}^{\ell }-J}\right)}^{-1}=\frac{J}{k_{B}J}{\left(\ln\frac{J+\varphi_{-}^{\ell }}{\varphi_{-}^{\ell }}\right)}^{-1},$$

which is at its minimum when $\varphi_{+}^{\ell }$ (or $\varphi_{-}^{\ell }$) reaches its minimum. For small flux, $\varphi_{+}^{\ell }$ (and $\varphi_{-}^{\ell }$) reaches its minimum also at maximal *E*_*j *_where concentration *x*_*ℓ*_is the lowest.

Note that with *J *given, the smallest conductance also corresponds to the largest |Δ*μ*|. In general, the rate-limiting step is not necessarily where the *k*'s are smallest. The most efficient situation for reaction system (44) is when all the conductances are equal. This result is analogous to the constant torque principle suggested for molecular motors [12,13,33].

### Flux-controlled and concentration-controlled biochemical networks

The analysis provided in the present paper poses the following biological question. When is a NESS of a biochemical network controlled by a constant flux injection and when is it controlled by a clamped concentration (chemical potential)? Clearly in real biological systems, the appropriate answer is a combination of both. Just as a real battery, having both finite internal resistance and conductance, in an electrical circuit is a combination of constant-current (zero internal conductance) and constant-voltage (zero internal resistance) ideal batteries, so too would a real biological system be a combination of constant flux injections and clamped concentrations [17]. Nevertheless, a brief discussion on this topic provides insights into the control of biochemical networks.

First, let's see what are the respective consequences of these two different types of "forcing" to a biochemical system. For a constant flux injection *J *to a simple unimolecular reaction in a NESS, we have the heat dissipation rate (*hdr*)

(47) $$hdr=-J\Delta \mu =k_{B}TJ\ln\left(1+\frac{J}{\varphi_{-}}\right).$$

This indicates that for the same injection flux *J *a reaction with larger *φ*_- _(i.e., conductance) dissipates a smaller amount of heat. For a constant clamped chemical potential Δ*μ*, we have

(48) $$hdr=\Delta \mu \varphi_{-}\left(1-e^{-\Delta \mu /k_{B}T}\right).$$

We note that *hdr *increases with *φ*_- _for the constant potential difference (concentration clamping) case. These results are analogous to an electric circuit in which the heat dissipation equals *I*^2^*R *and *U*^2^/*R*, respectively.

This observation leads us to the following hypothesis. In a biochemical network for biosynthesis, the fluxes are essential for supply and demand. Hence, such networks will in general have large conductances in order to minimize the energy waste and be efficient. On the other hand, in a biochemical network for cellular signaling, the concentrations are the essential signal. Thus, in this case the conductances are generally small in order to minimize the energy waste.

### Relation to the biochemical systems analysis

MCA shares an intimate relationship with the biochemical systems analysis (BSA) originally proposed by M.A. Savageau [21]. A critique of BSA can be found in [34]. BSA assumes that each metabolic reaction is catalyzed by an enzyme with rapid pseudo-steady-state enzyme-substrate complexes. This assumption leads to an explicit mathematical expression for the flux *J *in the enzymatic reaction as a rational function of the concentration of a substrate *x *[18], such that

(49) $$J=\frac{\alpha_{0}(x+a_{1})(x+a_{2})\cdots (x+a_{K})}{\beta_{0}(x+b_{1})(x+b_{2})\cdots (x+b_{L})},$$

where *K *≤ *L *due to substrate saturation or effector inhibition. The coefficients *a*_*i*_, *b*_*i*_, *α*_0_, and *β*_0 _contain the concentrations of all the other substrates. BSA provides insight to the properties of *a*_*i *_and *b*_*j*_. The network of metabolic reactions is then modeled [22] by a system of enzymatic reactions with a rate law given in the form of (49).

In terms of Eq. (49) the local elasticity-coefficient for the flux *J *can be written as

(50) $$\varepsilon =\frac{x}{x+a_{1}}+\frac{x}{x+a_{2}}+\cdots +\frac{x}{x+a_{K}}-\frac{x}{x+b_{1}}-\frac{x}{x+b_{2}}-\cdots -\frac{x}{x+b_{L}},$$

which also has the standard form of a Pade approximation [35]. Furthermore, BSA proposed [22] to approximate (49) by a power-law expression

(51) $$J\approx Cx_{1}^{\varepsilon_{1}}x_{2}^{\varepsilon_{2}}\cdots x_{N}^{\varepsilon_{N}},$$

which is essentially the integral form of the definition of local elasticity-coefficients.

In contrast, MCA assumes no specific rate laws for an enzymatic reaction except that the rate is linearly proportional to the enzyme concentration. This assumption is certainly valid for most biochemical reactions except when a substrate concentration is significantly less than that of an enzyme.

## Methods

### Solving the central, control-analysis equations

To demonstrate that the matrix, (**K ***ε*'), that appears in the central, control-analysis equations, i.e., Eqs. (24) and (36), is invertible, we note that since we are dealing with systems without conserved quantities, rank(**S**) = *N*. Furthermore, we note that the matrix **K**^*T*^**K **is invertible because **K **contains linearly independent columns, and because all columns of **K **are in the right nullspace of **S**, the matrix

$$\left(\begin{array}{l} S\\ K^{T} \end{array}\right)$$

is also invertible [23]. Therefore, we have

$$\begin{array}{l} \left(\begin{array}{cc} K & \varepsilon^{\prime } \end{array}\right)\left(\begin{array}{c} {\left(K^{T}K\right)}^{-1}K^{T}(I-R^{T}BS)\\ (\text{dg }x)(\text{dg }B)S \end{array}\right)\\ ={\left(\begin{array}{l} S\\ K^{T} \end{array}\right)}^{-1}\left(\begin{array}{l} S\\ K^{T} \end{array}\right)\left(\begin{array}{cc} K & \varepsilon^{\prime } \end{array}\right)\left(\begin{array}{c} {\left(K^{T}K\right)}^{-1}K^{T}(I-R^{T}BS)\\ (\text{dg }x)(\text{dg }B)S \end{array}\right)\\ ={\left(\begin{array}{l} S\\ K^{T} \end{array}\right)}^{-1}\left(\begin{array}{ll} 0_{N\times (M-N)} & S\varepsilon^{\prime }\\ K^{T}K & K^{T}\varepsilon^{\prime } \end{array}\right)\left(\begin{array}{c} {\left(K^{T}K\right)}^{-1}K^{T}(I-R^{T}BS)\\ (\text{dg }x)(\text{dg }B)S \end{array}\right)\\ ={\left(\begin{array}{l} S\\ K^{T} \end{array}\right)}^{-1}\left(\begin{array}{l} S\\ K^{T} \end{array}\right)=I. \end{array}$$

## Abbreviations

BCT: Biochemical Circuit Theory; BSA: Biochemical Systems Analysis; CMC: Correlation Metric Construction; FBA: Flux Balance Analysis; MCA: Metabolic Control Analysis; NESS: Nonequilibrium Steady-State; **A**: *N *× *N *linear stability matrix; **B**: *N *× *N *matrix = **A**^-1^; **K**: *M *× (*M *- *N*) loop (or nullspace) matrix (**SK **= **0**); **R**: *N *× *M *correlation matrix defined such that **A **= **SR**^*T*^; **S**: *N *× *M *stoichiometric matrix; **x**: *N*-dimensional vector of species' concentrations; **J**: *M*-dimensional vector of reaction fluxes; **Δ***μ*: *M*-dimensional vector of reaction potentials; ***ε*: ***M *× *N *local, elasticity-coefficient matrix; ***ε*: ***M *× *N *steady-state, elasticity-coefficient matrix; $\hat{C}$: *N *× *M *concentration, control-coefficient matrix; **C**: *M *× *M *flux, control-coefficient matrix; **C**^Δ*μ*^ : *M *× *M *biochemical potential, control-coefficient matrix; (dg **v**): If **v **is a vector, (dg **v**) denotes the diagonal matrix with the components of **v **along its diagonal. If **v **is a matrix, (dg **v**) denotes the diagonal matrix with only the diagonal components of **v **along its diagonal; *: Denotes steady-state; ': Distinguishes unscaled coefficients from scaled coefficients.

## Authors' contributions

HQ initiated this work. All authors contributed to and wrote the manuscript together. All authors read and approved the final manuscript.
